# Supplementary material for: Mutations in PCYT2 disrupt etherlipid biosynthesis and cause a complex hereditary spastic paraplegia
Source: Brain. 2019 Oct 22;142(11):3382–97. doi: 10.1093/brain/awz291 (PMC6821184; doi:10.1093/brain/awz291)
Supplement: awz291_Supplementary_Data [file awz291_supplementary_data.zip › awz291-Suppl_data/Supplementary_Data4.zip!/Supplementary information 3 - fibroblast lipidomics.zip!/Fibroblast lipidomics/Patient1 vs Patient2/Statistical plots and data/SM[t] species/Boxplots all.pdf]

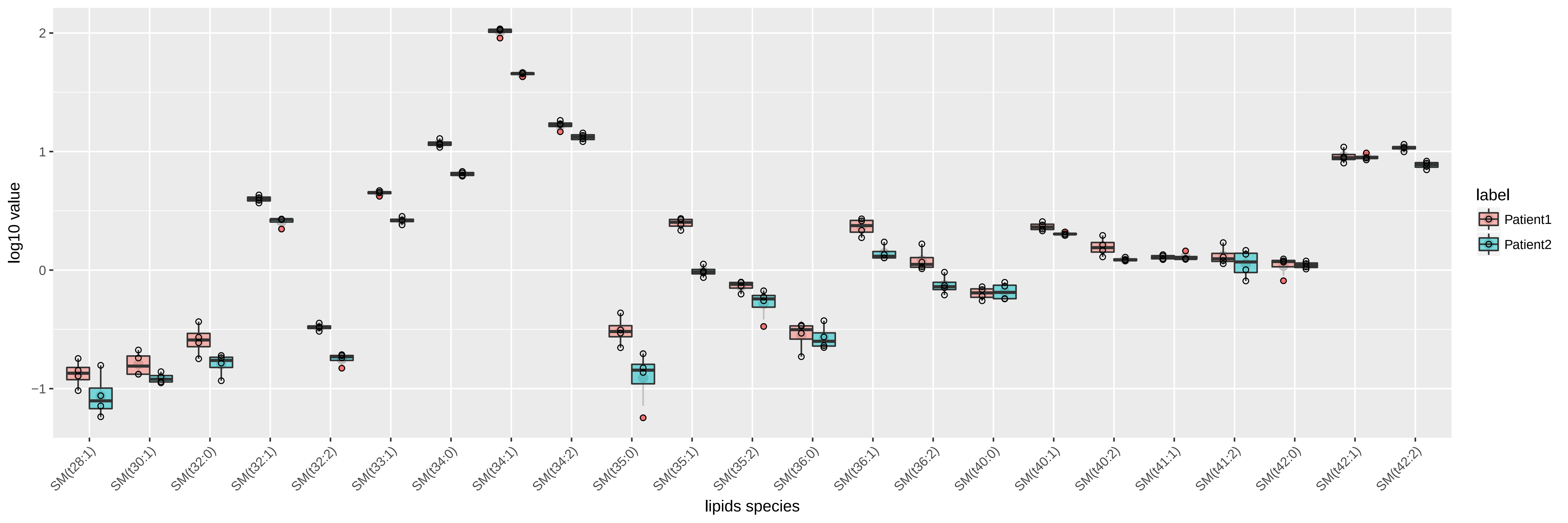

log10 value
2
1
0
−1
SM(t28:1)
SM(t30:1)
SM(t32:0)
SM(t32:1)
SM(t32:2)
SM(t33:1)
SM(t34:0)
SM(t34:1)
SM(t34:2)
SM(t35:0)
SM(t35:1)
SM(t35:2)
SM(t36:0)
SM(t36:1)
SM(t36:2)
SM(t40:0)
SM(t40:1)
SM(t40:2)
SM(t41:1)
SM(t41:2)
SM(t42:0)
SM(t42:1)
SM(t42:2)
lipids species
label
Patient1
Patient2
